# Widespread signatures of selection for secreted peptidases in a fungal plant pathogen

**DOI:** 10.1186/s12862-018-1123-3

**Published:** 2018-01-24

**Authors:** Parvathy Krishnan, Xin Ma, Bruce A. McDonald, Patrick C. Brunner

**Affiliations:** Plant Pathology, Institute of Integrative Biology, ETH Zurich, Universitätstrasse 2, -8092 Zurich, CH Switzerland

**Keywords:** Peptidases, Transcriptomics, Adaptive evolution, Neo-functionalization (NEO-F), Escape from adaptive conflict (EAC)

## Abstract

**Background:**

Fungal plant pathogens secrete a large arsenal of hydrolytic enzymes during the course of infection, including peptidases. Secreted peptidases have been extensively studied for their role as effectors. In this study, we combined transcriptomics, comparative genomics and evolutionary analyses to investigate all 39 secreted peptidases in the fungal wheat pathogen *Zymoseptoria tritici* and its close relatives *Z. pseudotritici* and *Z. ardabiliae*.

**Results:**

RNA-seq data revealed that a majority of the secreted peptidases displayed differential transcription during the course of *Z. tritici* infection, indicative of specialization for different stages in the life cycle. Evolutionary analyses detected widespread evidence of adaptive evolution acting on at least 28 of the peptidases. A few peptidases displayed lineage-specific rates of molecular evolution, suggesting altered selection pressure in *Z. tritici* following host specialization on domesticated wheat. The peptidases belonging to MEROPS families A1 and G1 emerged as a particularly interesting group that may play key roles in host-pathogen co-evolution, host adaptation and pathogenicity. Sister genes in the A1 and G1 families showed accelerated substitution rates after gene duplications.

**Conclusions:**

These results suggest widespread evolution of secreted peptidases leading to novel gene functions, consistent with predicted models of “escape from adaptive conflict” and “neo-functionalization”. Our analyses identified candidate genes worthy of functional analyses that may encode effector functions, for example by suppressing plant defenses during the biotrophic phase of infection.

**Electronic supplementary material:**

The online version of this article (10.1186/s12862-018-1123-3) contains suppl ementary material, which is available to authorized users.

## Background

Proteolysis, the enzymatic breakdown of proteins by peptidases, is an essential process in all organisms. While the main function of peptidases is protein turnover, peptidases also are key regulators of physiological processes including fertilization, embryogenesis, cell signaling, and immune responses and they play an important role in adaptation to stress [1]. Peptidases are substrate specific and are differentially expressed, both spatially and temporally [1].

In fungal pathogens, peptidases are known to affect spore formation and germination [2] and secreted peptidases may act as virulence factors [3–5]. Fungal peptidases can inactivate or modify protein components of the host defense machinery and ultimately suppress defense responses [6]. Some avirulence effectors are peptidases [7, 8] indicating that plants have evolved systems to recognize peptidases secreted by pathogens. Although earlier studies confirmed the contributions of peptidases to pathogenesis in *Botrytis cinerea* [9], *Fusarium culmorum* [10], *Endothia parasitica* [11, 12], *Glomerella cingulata* [13], and *Sclerotinia sclerotiorum* [14], little is known about their role in virulence for *Zymoseptoria tritici*.

*Zymoseptoria tritici* (synonym: *Mycosphaerella graminicola*) is an important fungal pathogen causing septoria tritici blotch on wheat*. Zymoseptoria tritici* is generally considered a hemibiotrophic fungus with three characteristic stages in its life cycle [15–17] (but see Sanchez et al. [18], for a different interpretation). During the initial biotrophic phase, the fungus grows slowly and asymptomatically for 10–15 days in the plant apoplast. The necrotrophic phase lasts for 1–3 days and is characterized by plant cell death and rapid fungal growth. During the final saprotrophic phase that lasts for several weeks, the fungus colonizes the dead plant tissue and forms reproductive structures [19].

Plant cell wall degrading enzymes (PCWDEs) constitute another important group of secreted proteins that facilitates pathogen invasion. PCWDEs or their degradation products can also act as elicitors of plant defense responses. The genome of *Z. tritici* encodes a reduced number of PCWDE genes compared to other fungal plant pathogens. Based on this and other observations, Goodwin et al. [20] proposed a biphasic mechanism of stealth pathogenesis in *Z. tritici*. According to this hypothesis, the plant nutrients obtained during the extended biotrophic phase result mainly from degradation of proteins and/or low molecular weight molecules present in the plant apoplast rather than from the degradation of carbohydrates by PCWDEs. Brunner et al. [16] provided support for this hypothesis by showing that only a few PCWDEs are expressed during the biotrophic phase. However, other hypotheses proposed that peptidases that are up-regulated during the biotrophic phase could act as effectors, for example by suppressing host defenses through degradation of plant-derived pathogenesis-related proteins [21].

Here, we explored these competing hypotheses by combining comparative genomics, transcriptomics and selection analyses to investigate the evolutionary patterns and signatures of selection acting on secreted peptidases in *Z. tritici*.

## Methods

### Comparative genomics

The annotated genome of *Zymoseptoria tritici* isolate IPO323 from JGI (https://genome.jgi.doe.gov/Mycgr3/Mycgr3.info.html; *Mycosphaerella graminicola* v2.0; [20]) was used as a reference throughout this study. Thirty-nine peptidases are predicted to be secreted in *Z. tritici* [22]. Complete sequences of all 39 genes were retrieved from the JGI annotation and the Basic Local Alignment Search Tool (BLAST) [23] was used to identify their homologs in 29 new whole-genome assemblies of *Z. tritici* [24]. Five genomes of *Z. pseudotritici* and four genomes of *Z. ardabiliae* (NCBI BioProject PRJNA63131), the closest known relatives of *Z. tritici* [25] were included as outgroups. Sequences of each reference gene from IPO323 were mapped to genomic scaffolds of the re-sequenced *Zymoseptoria* spp. isolates using BLASTN. Exon regions of identified homologs were aligned on the amino acid level using ClustalW multiple sequence alignment [26]. All sequences were visually inspected for ambiguous alignment of coding or flanking regions. As a comparison, an equal number of non-secreted peptidases were chosen randomly from the JGI annotations of reference isolate IPO323. These peptidases had i) no signal peptide as determined by SignalP [27] ii) were expressed during at least one stage of infection according to RNAseq data, and iii) had complete annotations including start and stop codons. The sequences for the non-secreted peptidases were extracted from the same 29 re-sequenced whole-genome assemblies of *Z. tritici*.

### Transcriptomics

RNA-seq [28] was used to quantify gene expression during the three different stages of the *Z. tritici* life cycle. We retrieved expression profiles for all peptidases from an earlier RNA-seq experiment [29]. Briefly, *Z. tritici* strain ST99CH3D7 was inoculated onto wheat seedlings. Infected leaves were sampled at 7, 14 and 28 days post inoculation (dpi), corresponding to the biotrophic, necrotrophic and saprotrophic stages of the fungal life cycle, respectively. RNA sequencing was performed on an Illumina HiSeq 2500 using paired end reads at 2 × 125 bp. Raw RNA-seq reads were trimmed using Trimmomatic v. 0.33 [30]. Trimmed reads were aligned to the *Z. tritici* reference genome retrieved from Ensembl release 26 [31] (accessed May 2015) and transcriptome [32]. To calculate the gene counts HTSeq v0.6.1 [33] was used. Differential gene expression analysis was performed using the R package EdgeR version 3.2.3 [34].

TMM (trimmed mean of M-values) normalization is an effective method for estimating relative RNA levels in RNA-seq experiments [35]. Mean TMM normalized RPKM (reads per kilobase per million mapped reads) and mean TMM normalized log_2_ CPM (counts per million mapped reads) were calculated for each peptidase. Benjamin-Hochberg false discovery rates (FDR) were calculated to identify significantly up-regulated genes in different stages of the life cycle. Hereafter, we define life cycle stage-specific expression as a ≥ 5-fold difference in RPKM values compared to the expression values observed during the other stages, with an FDR adjusted *p*-value ≤0.05. Mean TMM normalized log_2_ CPM and RPKM values were calculated for all 39 secreted peptidases. Mean normalized log_2_ CPM values were transformed using Z scores. Means of these Z-score transformed transcription values (normalized log_2_ CPM) of each peptidase gene family were plotted to compare expression levels at different life cycle stages.

### Selection analyses

Average dN/dS ratios for the secreted and non-secreted peptidase genes were calculated from pairwise sequence comparisons using DnaSP [36]. In addition, we conducted codon-based selection analyses using the PAML 4.8 program package (Phylogenetic Analysis by Maximum Likelihood) [37, 38]. A major drawback of dN/dS tests is that they cannot make a clear distinction between positive selection and recombination. PAML assumes that the sequences in the alignment are related by a single phylogeny, however this is not true whenever a recombination event occurs [39]. To overcome this limitation we performed PAML analyses on non-recombining trees generated with RDP4 (Recombination Detection Program v.4.7) [40] and the implemented version 8 of RAxML (Randomized Axelerated Maximum Likelihood; [41]), using the default setting.

We performed the selection analyses at different hierarchical levels. First, we used the implemented “site model” to determine overall signatures of selection for each gene. This approach consists of comparing likelihood estimates obtained with different evolutionary models. Two different models that allowed ω (dN/dS ratio, the ratio of non-synonymous mutations per non-synonymous sites to synonymous mutations per synonymous sites) to vary across codons were used to perform a site-by-site detection of selection [42]. The neutral model M7 has two codon site classes that allow ω to vary between 0 and 1 (deleterious and neutral mutations, respectively) and the selection model M8 has an additional site class compared to M7, allowing ω to be > 1 (signifying diversifying selection). Site models M7 and M8 were fitted to our data and likelihood estimates were compared for significant differences using hierarchical likelihood ratio tests (LRT) (Nielsen & Yang, 1998).

In the second analysis we implemented the PAML “branch model” to identify signatures of selection acting on a specific lineage of the phylogenetic tree [43, 44]. We tested whether the selection pressure acting on the branch leading to *Z. tritici* (i.e. the foreground branch) differed from the branches leading to its sister species (i.e. the background branches). The branch model allows ω to vary on the foreground branch, while the ω value for the background branches is fixed. Selection analyses were performed by comparing the branch model, with the M0 model that estimates one ω value for the entire tree topology.

Following gene duplication events, sister genes can evolve at different evolutionary rates [45, 46]. Therefore, we used also the PAML branch model approach to compare signatures of selection acting on the two branches after a gene duplication event. We restricted this analysis to the A1 and G1 peptidase families because all members of these families showed significant signatures of selection in our previous analyses. To avoid the comparison of non-homologous sequences, PAML analyses were conducted on reduced gene trees, containing only members of the same gene family. To make our approach more conservative, all sites with ambiguous character states and alignment gaps were removed prior to the analyses using the “clean-data” option in PAML. Since our main interest was the identification of candidate genes encoding effector functions, we restricted the detailed selection analyses to the secreted peptidases.

## Results

### Gene homologies

Sequences of all 39 secreted peptidases were retrieved from the reference genome of *Z. tritici* IPO323. These were classified into 9 clans and 11 families according to the MEROPS database [47]. Homologs for all peptidases were identified in all 29 re-sequenced genomes of *Z. tritici*, with an average amino acid identity of 99%. Orthologous genes for 37 peptidases were identified in *Z. pseudotritici* (93.2% average amino acid identity) and 35 were identified in *Z. ardabiliae* (91.2% average amino acid identity; Additional file 1: Table S1). All 39 secreted peptidases identified in *Z. tritici* were included in the analyses along with their homologs in *Z. pseudotritici* and *Z. ardabiliae* as outgroups. Only one sister species was included if the corresponding homolog was not found in both (Additional file 1: Table S1).

### Transcriptomics

**Fig. 1:**
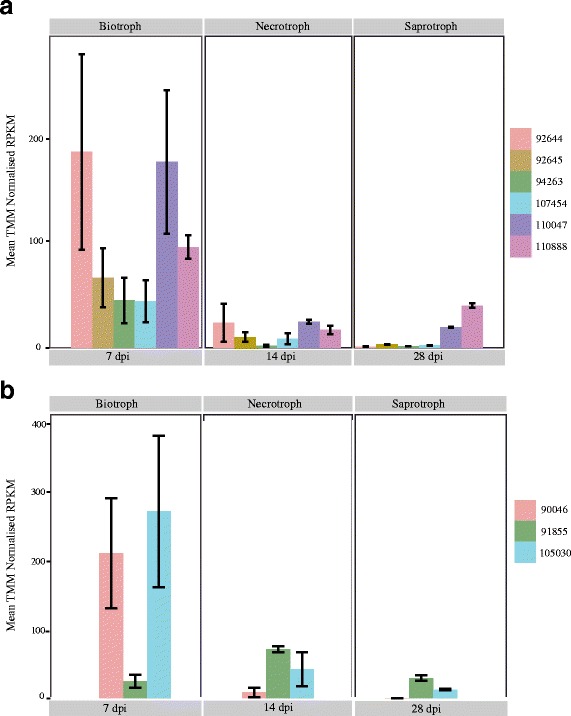
Expression patterns of secreted peptidases in gene families A1 (panel **a**) and G1 (panel **b**) in *Zymoseptoria tritici.* The x-axis shows TMM (trimmed mean of M values) normalized RPKM values (reads per kilo base per million mapped reads) from RNA-seq experiments in *Z. tritici.* The gene expression bars represent the average from three biological replicates and the error bars are the standard error of the mean. Prot. ID is the protein identification number from the JGI database for the reference strain IPO323. Biotroph, necrotroph and saprotroph correspond to the three life cycle stages of *Z. tritici* measured at 7 days post inoculation (dpi), 14 dpi and 28 dpi, respectively. All the genes except for the one encoding Prot. ID-91855 were ≥5-fold upregulated during the biotrophic phase, with a FDR-corrected *p* value ≤0.05

**Fig. 2:**
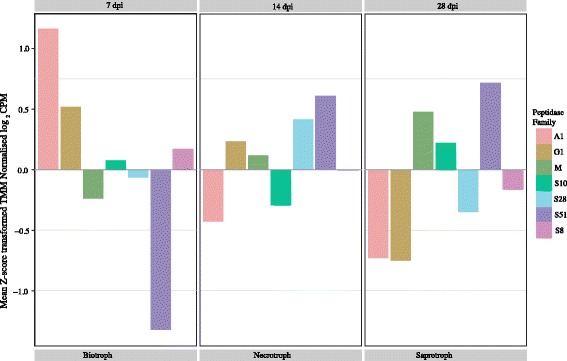
Differential expression of peptidase families during different life cycle stages. Means of Z-score transformed TMM Normalized log _2_ CPM values for each gene family. Biotroph, necrotroph and saprotroph correspond to the three investigated life cycle stages of *Zymoseptoria tritici* measured at 7 days post inoculation (dpi), 14 dpi and 28 dpi, respectively

We used RNA-seq to investigate transcription of each peptidase gene during the course of the *Z. tritici* infection cycle. The expression analyses are summarized in Additional file 1: Tables S2 and S3. Twenty-two of the 39 peptidases (56%) displayed life cycle stage-specific expression. For example, all six members of the A1 family were highly expressed during the biotrophic phase and significantly down-regulated during the other phases (Fig. 1a). Similarly, two of the three members of the G1 family (Prot. ID-90046 and Prot. ID-105030) showed life cycle stage-specific expression and were highly up-regulated during the biotrophic phase (Fig. 1b). The mean TMM normalized RPKM values also indicated higher overall transcription for the A1 and G1 families during the biotrophic phase (Fig. 2) compared to the other phases of infection.

### Selection analyses

Average dN/dS ratios calculated from pairwise sequence comparisons are shown in the Additional file 1: Table S4 and Fig. S1. The secreted peptidases had significantly higher dN/dS ratios compared to the non-secreted peptidases (Wilcoxon rank-sum test; *p* = 0.03). Several secreted peptidases had dN/dS > 1. In contrast, all of the analyzed non-secreted peptidases had dN/dS < 1.

**Fig. 3:**
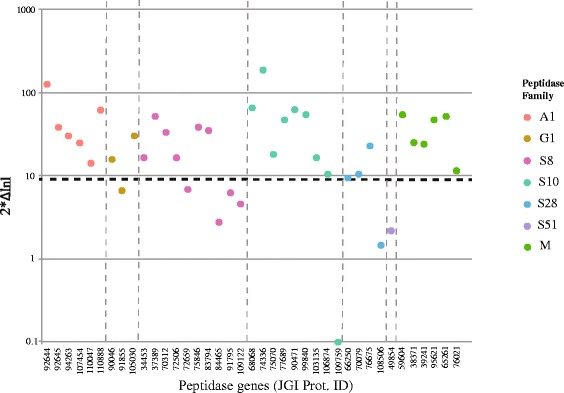
Scatter plot indicating the 2*Δlnl values resulting from the PAML site model analyses for all 39 secreted peptidases. The black dashed line (--) indicates the threshold value for a significant likelihood ratio test (*p* < 0.01). Values above this line are indicative of adaptive (accelerated) evolution. The y-axis is represented using a logarithmic scale. The colors represent the major families of secreted peptidases

All peptidases showed high nucleotide variability within and/or between species. PAML analyses detected significant deviations from neutral expectations of codon evolution for 28 out of 39 peptidases, indicating widespread adaptive evolution for this protein family (Fig. 3). For example, the LRT test was highly significant for all six peptidases in the A1 family, suggesting diversifying selection at several codon sites in these genes (Additional file 1: Table S5). An example of a phylogenetic tree used for the PAML branch model analyses to infer signatures of selection for the *Zymoseptoria tritici* lineage is shown in Additional file 1: Figure S2. It has to be mentioned here that unusually long branches in a phylogenetic tree can be indicative of non-homology among sequences. The long basal branches in the phylogenetic reconstruction of the peptidase families (Fig. 5) might therefore not reflect true evolutionary relationships by a common ancestor, but rather convergent evolution towards a similar function.

**Fig. 4:**
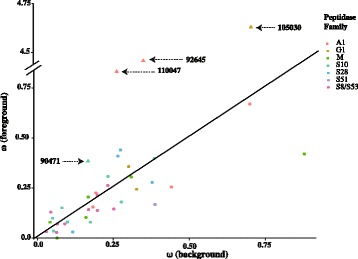
Scatter plot indicating the dN/dS ratios (ω) estimated with the PAML branch model analyses for all 39 secreted peptidases in *Zymoseptoria* species. ω values of the background branch (*Z. pseudotritici* and *Z. ardabiliae*) are represented on the x-axis and the ω values of the foreground branch (*Z. tritici*) are represented on the y-axis. The triangle symbol (Δ) indicates genes under significantly accelerated evolution (Prot. IDs-92645, 10047, 105030 and 90471). The colors correspond to the major classes of secreted peptidases

**Fig. 5:**
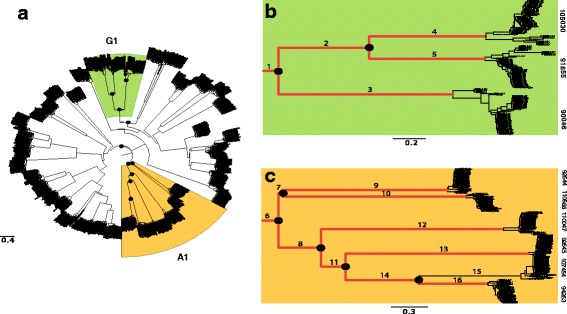
Phylogenetic reconstruction used in the PAML analyses to infer branch-specific signatures of selection after gene duplications (indicated by black dots) in the peptidase families G1 and A1. A. maximum likelihood tree depicting the evolutionary relationship of all secreted peptidases in *Zymoseptoria tritici*, *Z. pseudotritici* and *Z. ardabiliae*. B. Enlarged view of peptidase family G1. C. Enlarged view of peptidase family A1. Analyzed branches are indicated by numbers that correspond to Additional file 1: Table A7. Red branches denote significant accelerated evolution. Numbers on the right are protein identification numbers according to Additional file 1: Table S1. The trees are drawn to scale, with branch lengths in units of nucleotide substitutions per site

The PAML branch model detected four genes in the *Z. tritici* lineage evolving significantly faster than in the sister species (Prot. IDs-92645, 110047, 105030, 90471; Fig. 4, Additional file 1: Table S6). We also found strong evidence for differential selection acting during the evolutionary history of the members of families A1 and G1. Our analyses of sister genes resulting from gene duplication events showed an increase in evolutionary rates after the first two duplication events leading to the A1 and G1 families (Additional file 1: Table S7; Fig. 5). Subsequently, the genes of the G1 family exhibited diversifying selection on all branches following each gene duplication, suggesting continuous adaptive evolution. In the A1 family, we found a more complex pattern comprising diversifying, relaxed and purifying selection.

## Discussion

The *Zymoseptoria* species complex provides a powerful model to investigate the evolutionary processes affecting genes involved in host-pathogen coevolution. Several recent studies analyzed the transcription profiles of secreted proteins in *Zymoseptoria tritici* during a complete infection cycle on wheat [16, 29, 48]. Brunner et al. [16] showed that genes encoding different members of PCWDE families displayed life cycle stage-specific expression, with several genes exhibiting different patterns of selection. In this study, we took a similar approach, combining transcriptomics data with selection analyses to infer the evolutionary processes operating on secreted peptidases. We detected widespread signatures of adaptive evolution. Peptidases classified into families A1 and G1 of the MEROPS database emerged as a particularly interesting group because these two families were preferentially expressed during the biotrophic phase and displayed higher substitution rates than expected under neutral evolution. This allowed us to formulate hypotheses regarding the potential role of secreted peptidases in *Z. tritici,* and to identify candidate genes for subsequent functional studies and validation.

Other plant pathogenic fungi with biotrophic phases of infection were found to locate highly selected pathogenicity genes in repeat-rich regions (*Leptosphaeria maculans* [49]) or in clusters (*Ustilago maydis* [50]). Intriguingly, we found that two pairs of genes (92644 and 92645 on chromosome 4 and 110047 and 105030 on chromosome 7) were located close to each other in telomeric regions rich in transposable elements (TEs) (Additional file 1: Table S8). A previous study showed that these TE-rich regions in *Z. tritici* are highly dynamic and are enriched in genes encoding putative effectors [51]. A genome-wide analysis of the smut fungus *Melanopsichium pennsylvanicum* reported that putative secreted effectors showed a higher proportion of genes under diversifying selection than non-effector candidates [52]. The same study also concluded that the host-jump from monocots to dicots in *M. pennsylvanicum* was associated with the loss of putative effector genes. In *Z. tritici*, the loss or gain of genes is facilitated by TE-mediated chromosomal rearrangements [51]. The genome of *Z. tritici* also contains a variable number of accessory chromosomes that further facilitate gene gains and losses. None of the investigated peptidases were located on accessory chromosomes and all investigated isolates of *Z. tritici* contained the full set of secreted peptidases. However, we could not detect orthologs of two peptidases in *Z. pseudotritici*, and four orthologs were missing from *Z. ardabiliae* (Additional file 1: Tables S1 and S8). Thus, in contrast to *M. pennsylvanicum*, we observed a gain in peptidases in the pathogen lineage that adapted to domesticated wheat, compared to its ancestors found on wild grasses. Notably, one of these missing orthologs (92645) was identified as one of four candidate effector genes in *Z. tritici*.

The RNA-seq analysis revealed that 22 out of 39 secreted peptidases showed expression patterns that were life cycle stage-specific, with 12 genes being up-regulated ≥5-fold during the biotrophic phase. Strikingly, all six members of the A1 family were up-regulated during the biotrophic phase. Higher expression of peptidases during the biotrophic phase is in accordance with the hypothesis of stealth pathogenesis proposed by Goodwin et al. [20]. According to this hypothesis, *Z. tritici* obtains nutrients during an extended biotrophic phase mainly through degradation of plant proteins located in the apoplast. However, this remains to be demonstrated. An alternative interpretation is that peptidases that are highly expressed during the biotrophic phase function as effectors, for example by suppressing apoplastic immunity through degrading plant-derived pathogenesis-related proteins ([21] and references therein). We observed that 10 out of the 12 secreted peptidases that were up-regulated during the biotrophic phase exhibited significantly accelerated evolution. On average, the dN/dS ratio was significantly higher for the secreted peptidases (Additional file 1: Table S4 and Figure S1). However, we investigated only a sub-set of the annotated non-secreted peptidases in *Z. tritici* and we consider it likely that some non-secreted peptidases are also under strong diversifying selection. For example, following a host switch, a pathogen’s peptidases and other nutrition-related proteins will need to adapt to novel host substrates. This will likely be detectable as elevated genetic diversity at these loci if the switch occurred relatively recently.

Our finding that the majority of secreted peptidases in *Z. tritici* display signatures of diversifying selection is indicative of a widespread accelerated evolution reflecting processes of dynamic adaptation for these molecules. It remains to be tested whether these signatures of adaptation directly reflect processes of host-parasite interactions such as gene-for-gene coevolution. However, diversifying selection in plant pathogens has been detected for many genes involved in arms races with their hosts, particularly for genes encoding effector proteins [25, 53, 54]. The host deploys specialized proteins that have evolved to detect the pathogen and to activate subsequent defense responses. The pathogen in turn co-evolves to avoid host recognition and to suppress the host’s defense responses. Some of the effectors secreted by plant pathogens are peptidases that can alter key proteins involved in host defense [55, 56]. We identified four peptidase genes under diversifying selection that were up-regulated during the biotrophic stage. We hypothesize that these secreted peptidases are effectors that suppress apoplastic immunity by breaking down plant-derived pathogenesis-related proteins during the biotrophic phase [21]. This hypothesis is based on earlier reports that wheat defense responses against fungal infections involve the production of peptidase inhibitors. For example, *Botrytis cinerea* is inhibited by the Bowman-Birk trypsin inhibitor found in wheat kernels [57] and serine peptidases secreted by *Z. tritici* are inhibited by the germin-like peptidase inhibitor (GLPI) found in the wheat leaf apoplast [58].

Four out of the 39 secreted peptidases showed a significant increase in dN/dS along the branch leading to *Z. tritici*, suggesting accelerated evolution of these proteins during the adaptation from wild grasses onto wheat (Additional file 1: Table S5). However, an increase in dN/dS can result from diversifying selection or a relaxation of purifying selection. We therefore conducted additional LRT tests to distinguish between these alternatives. The LRT tests confirmed that one gene (Prot. ID-90471) was under significant diversifying selection, while the other three genes were under relaxed purifying selection (Additional file 1: Table S5). Zhang et al. [59] demonstrated that both diversifying selection and relaxation of purifying selection are key mechanisms leading to functional divergence of duplicated genes. A weakening of selection or a constraint in selection could lead to the condition of relaxed selection [60]. We postulate that in this case the relaxation of selection is due mainly to the host shift from wild grasses to domesticated wheat.

Gene duplication is an important source of evolutionary novelty and is thought to play a central role in generating the raw material required for adaptive evolution [61]. Gene duplication may help an organism to successfully adapt to a changing environment, including the extreme case of host/pathogen co-evolution [62]. The most common outcomes after gene duplication are non-functionalization, sub-functionalization and neo-functionalization of the sister genes [63]. Non-functionalization is the process of accumulation of mutations in a duplicated gene that makes the sister gene non-functional. For example, in the plant pathogen *Leptosphaeria maculans,* repeat-induced point mutation led to the emergence of non-functional effector alleles that can evade recognition by RLM6 immune receptors [64]. Under the scenario of sub-functionalization, the expression pattern and function of the ancestral gene is shared between sister genes after the duplication. For the case of neo-functionalization (NEO-F), one of the sister genes is free to accumulate mutations and acquire a new function while the other gene maintains its ancestral function [65]. Skamnioti et al. [66] presented evidence for sub-functionalization and neo-functionalization in the plant pathogen *Pyricularia grisea* based on conserved or novel expression profiles in phylogenetically related cutinase genes. Recent studies proposed an additional evolutionary model called “escape from adaptive conflict” (EAC) [45, 67]. In this scenario the ancestral single copy gene is selected to perform a novel function in addition to its primary function, leading to constraints for optimization of each function. Duplication of this gene helps to resolve the conflict by allowing each sister gene to specialize to perform either the ancestral or the novel function after gene duplication [67]. Codon-based models can be used to distinguish among all of these evolutionary scenarios. For example, in the case of EAC, both duplicate copies will undergo diversifying selection, whereas in NEO-F only one of the sister genes will undergo diversifying selection while the other gene will undergo purifying selection to maintain and optimize the ancestral function [45].

The A1 and G1 peptidase families showed particularly interesting patterns of evolution and transcription in *Z. tritici*. Our transcriptomic studies showed that all members of the A1 aspartic peptidase family and most members of the G1 family were up-regulated during the asymptomatic biotrophic phase. In addition, all but one member of the A1 and G1 families were under adaptive evolution. Previous studies showed that fungi have a relatively higher complement of aspartic peptidases than other eukaryotes. Aspartic peptidases show high diversity and interspecific clustering and are believed to have undergone a process of birth and death during the course of evolution [68]. Thus, we investigated in more detail the evolutionary history of the sister genes in A1 and G1 (Table 1).

**Table 1:** Categorization of secreted peptidases in the A1 and G1 families into different evolutionary scenarios. Life cycle stages showing peaks of expression are indicated as B (biotrophic) N (necrotrophic) and S (saprotrophic), respectively

MEROPS	JGI	Peak of gene expression	Site model	Branch model		Branch model		Evolution Model
Family	ProtID.			*Z. tritici* lineage		Gene duplication		
			*P* value	ω > 1	*P* value	ω > 1	*P* value	
A1	92644	B	S	No	NS	Yes	S	EAC
A1	92645	B	S	No	S	No	S	EAC
A1	94263	B	S	No	NS	Yes	S	NEO-F
A1	107454	B	S	No	NS	No	NS	Conservation
A1	110047	B	S	No	S	Yes	S	EAC
A1	110888	B	S	No	NS	Yes	S	EAC
G1	90046	B	NS	No	NS	Yes	S	EAC
G1	91855	N	S	No	NS	Yes	S	EAC
G1	105030	B	S	Yes	S	Yes	S	EAC

EAC; escape from adaptive conflict. NEO-F; neo-functionalization. NS; non-significant, S; significant

We tested all genes in the A1 and G1 peptidase families for signatures of accelerated evolution following gene duplications. We observed that the basal branches 1 and 6 (Fig. 5) are under significant diversifying selection with a very high ω value (999.000), consistent with a burst of evolution following the first duplication event leading to the A1 and G1 families. In the G1 family, all branches emerging from the subsequent gene duplication events were under significant diversifying selection, for example Prot. ID-105030 and Prot. ID-91855 (branches 4 and 5 in Fig. 5). We also observed differences in expression pattern between these sister genes. The gene encoding Prot. ID-105030 was highly expressed during the biotrophic phase, while the gene encoding Prot. ID-91855 was highly expressed during the necrotrophic phase (Fig. 1b). This suggests that members of the G1 family are constantly evolving to acquire new functions, consistent with the EAC evolutionary model [45].

All members of the A1 peptidase family, with the exception of the gene encoding Prot. ID-107454, also displayed an accelerated evolution after each duplication event. Their pattern of evolution differed from the pattern observed in G1, however, by including both diversifying and relaxed purifying selection after gene duplication events. We consider this a special case of EAC because both diversifying selection and relaxation of purifying selection can lead to functional divergence of duplicated genes [59]. The signature of neo-functionalization was also observed in the A1 peptidase family. The gene encoding Prot. ID-107454 was under purifying selection, maintaining its ancestral function. In contrast, its sister gene encoding Prot. ID-94263 was under diversifying selection, suggesting that this gene is evolving towards attaining a new function.

Until now, very few studies have shown signatures of diversifying selection on genes encoding peptidases secreted by fungi. Li et al. [69] found signatures of diversifying selection on genes encoding subtilisin-like serine peptidases in nematode-trapping fungi. Comparing genomes among several species of smut fungi, Sharma et al. [51] reported that putative secreted effector proteins showed a higher proportion of genes under diversifying selection than non-effector candidates. Here we report the first population genetic analysis of genes encoding peptidases in plant pathogenic fungi and demonstrate widespread signatures of diversifying selection. Additional functional experiments using gene knockouts or overexpression lines will be needed to test our hypothesis that peptidases play key roles in host pathogen co-evolution, host adaptation and pathogenicity in *Z. tritici*. Our analyses suggest that functional studies should focus first on members of the G1 and A1 peptidase families as potential candidate effector genes that suppress apoplastic immunity during the biotrophic phase of wheat infection.

## Additional file

Additional file 1: Widespread signatures of selection for secreted peptidases in a fungal plant pathogen. (DOCX 23507 kb)
