# Supplementary material for: Widespread signatures of selection for secreted peptidases in a fungal plant pathogen
Source: BMC Evol Biol. 2018 Jan 24;18:7. doi: 10.1186/s12862-018-1123-3 (PMC5784588; doi:10.1186/s12862-018-1123-3)
Supplement: Additional file 1: — Widespread signatures of selection for secreted peptidases in a fungal plant pathogen. (DOCX 23507 kb) [file 12862_2018_1123_MOESM1_ESM.docx]

Additional file 1.

**Table S1:** Identification of homologous and orthologous gene sequences encoding secreted peptidases in 38 whole genome assemblies of *Zymoseptoria tritici* and its closest relatives *Z. pseudotritici* and *Z. ardabiliae*. BLAST searches used search algorithms of > 50% amino acid identity with respect to 39 annotated secreted peptidases from the *Z. tritici* reference IPO323 genome. The number of amino acids and average amino acid identity (AA_ID) are given with respect to IPO323. Genes are listed according to the MEROPS classification into clans and families. Protein identification numbers (Prot. ID) follow the Joint Genome Institute (JGI) annotations.

| MEROPS | MEROPS | JGI | IPO323 |  | *Z.tritici* | (N=29) |  |  | *Z. pseudotritici* | (N=5) |  |  | *Z. ardabiliae* | (N=4) |
| --- | --- | --- | --- | --- | --- | --- | --- | --- | --- | --- | --- | --- | --- | --- |
| CLAN | Family | ProtID | amino acids |  | amino acids | % AA_ID |  |  | amino acids | % AA_ID |  |  | amino acids | % AA_ID |
|  |  |  |  |  |  |  |  |  |  |  |  |  |  |  |
| AA | A1 | 92644 | 460 |  | 460 | 98.13 |  |  | 460 | 87 |  |  | 460 | 85 |
|  | A1 | 92645 | 489 |  | 489 | 99.17 |  |  | 487 | 89 |  |  | ND | ND |
|  | A1 | 94263 | 459 |  | 459 | 98.9 |  |  | 459 | 92 |  |  | 461 | 91.5 |
|  | A1 | 107454 | 478 |  | 478 | 98.41 |  |  | 478 | 92 |  |  | 479 | 91 |
|  | A1 | 110047 | 441 |  | 441 | 99 |  |  | 445 | 84 |  |  | 445 | 83 |
|  | A1 | 110888 | 534 |  | 534 | 98.72 |  |  | 534 | 92.6 |  |  | ND | ND |
| GA | G1 | 90046 | 250 |  | 250 | 99.17 |  |  | 250 | 95.6 |  |  | 250 | 80 |
|  | G1 | 91855 | 253 |  | 253 | 98.96 |  |  | 256 | 92 |  |  | 254 | 91 |
|  | G1 | 105030 | 269 |  | 269 | 98.67 |  |  | 269 | 76 |  |  | 276 | 74 |
| SB | S8/S53 | 34453 | 392 |  | 392 | 99.31 |  |  | 392 | 97 |  |  | 392 | 95 |
|  | S8/S53 | 37389 | 627 |  | 627 | 99 |  |  | 627 | 91 |  |  | 627 | 89 |
|  | S8/S53 | 70312 | 507 |  | 507 | 99 |  |  | 507 | 99 |  |  | 507 | 99 |
|  | S8/S53 | 72506 | 651 |  | 651 | 99.32 |  |  | 651 | 98 |  |  | 651 | 96.5 |
|  | S8/S53 | 72659 | 400 |  | 400 | 99.2 |  |  | 400 | 98.8 |  |  | 400 | 98 |
|  | S8/S53 | 75846 | 608 |  | 608 | 99.14 |  |  | 608 | 98.6 |  |  | 608 | 98 |
|  | S8/S53 | 83794 | 593 |  | 593 | 99 |  |  | 593 | 98 |  |  | 569 | 84.5 |
|  | S8/S53 | 84465 | 660 |  | 660 | 99.93 |  |  | 660 | 77 |  |  | 660 | 90 |
|  | S8/S53 | 91795 | 275 |  | 275 | 99.42 |  |  | 419 | 96 |  |  | 419 | 94.5 |
|  | S8/S53 | 109122 | 358 |  | 358 | 99.28 |  |  | 358 | 96.6 |  |  | 358 | 90 |
| SC | S10 | 68068 | 578 |  | 578 | 99 |  |  | 581 | 91 |  |  | 588 | 86 |
|  | S10 | 74336 | 655 |  | 655 | 98.36 |  |  | 655 | 95.4 |  |  | 655 | 96 |
|  | S10 | 75070 | 506 |  | 506 | 99.36 |  |  | 523 | 95.4 |  |  | 524 | 94.5 |
|  | S10 | 77689 | 572 |  | 572 | 99 |  |  | 623 | 88.6 |  |  | 621 | 88.7 |
|  | S10 | 90471 | 619 |  | 619 | 98.96 |  |  | 619 | 87 |  |  | 619 | 86 |
|  | S10 | 99840 | 642 |  | 642 | 98.82 |  |  | 642 | 96 |  |  | 642 | 95.5 |
|  | S10 | 103135 | 549 |  | 549 | 99.14 |  |  | 549 | 99 |  |  | 549 | 99 |
|  | S10 | 106874 | 545 |  | 545 | 99.0 |  |  | 545 | 97 |  |  | 544 | 92.5 |
|  | S10 | 109759 | 546 |  | 546 | 99.93 |  |  | 546 | 99 |  |  | 546 | 98 |
|  | S28 | 66250 | 560 |  | 560 | 99.08 |  |  | 560 | 94 |  |  | 560 | 93 |
|  | S28 | 70079 | 528 |  | 528 | 98.67 |  |  | 528 | 91 |  |  | 525 | 88 |
|  | S28 | 76675 | 529 |  | 529 | 99.45 |  |  | 528 | 87 |  |  | 536 | 91 |
|  | S28 | 108506 | 543 |  | 543 | 99.03 |  |  | 543 | 98 |  |  | ND | ND |
| PC | S51 | 49854 | 298 |  | 298 | 99.53 |  |  | ND | ND |  |  | 298 | 92 |
| MC | M14 | 59604 | 426 |  | 426 | 98.47 |  |  | 426 | 97 |  |  | 426 | 97 |
| MA | M3 | 38371 | 742 |  | 742 | 99.56 |  |  | 742 | 93 |  |  | ND | ND |
| MA | M35 | 39241 | 365 |  | 365 | 99.13 |  |  | ND | ND |  |  | 365 | 88 |
| MH | M28 | 95621 | 656 |  | 656 | 97.71 |  |  | 656 | 96 |  |  | 656 | 93 |
| MH | M28 | 65261 | 552 |  | 552 | 98.89 |  |  | 552 | 99 |  |  | 549 | 97 |
| MA | M43 | 76021 | 279 |  | 279 | 99 |  |  | 279 | 95 |  |  | 280 | 88 |
|  | Average |  |  |  |  | 99.02102564 |  |  |  | 93.20540541 |  |  |  | 91.23428571 |

**Table S2:** Transcription data for 39 secreted peptidases of *Zymoseptoria tritici* listed according to the MEROPS classification system into clans and families. Prot. ID corresponds to protein identification number from the Joint Genome Institute (JGI). For each of the three lifecycle stages (biotroph, necrotroph and saprotroph) RNA-seq reads of biological replicates are represented as TMM normalized RPKM values and visualized as a heat-map formatted using a color scale of red to green (lowest to highest RPMK values). TMM normalized RPKM values are reported for each life cycle stage based on three biological replicates. Average RPKM values corresponding to each life cycle specific stage and standard deviations are also reported.

| MEROPS | MEROPS Family | JGI | 7 days post inoculation | | | 14 days post inoculation | | | 28 days post inoculation | | |  | 7 days post inoculation | | 14 days post inoculation | | 28 days post inoculation | |
| --- | --- | --- | --- | --- | --- | --- | --- | --- | --- | --- | --- | --- | --- | --- | --- | --- | --- | --- |
| CLAN |  | Prot ID | D7_R1 | D7_R2 | D7_R3 | D13_R1 | D13_R2 | D13_R3 | D28_R1 | D28_R2 | D28_R3 |  | Average RPKM | SD | Average RPKM | SD | Average RPKM | SD |
| AA | A1 | 92644 | 341.12 | 143.14 | 72.68 | 10.26 | 54.72 | 5.77 | 0.38 | 1.15 | 0.72 |  | 185.65 | 139.18 | 23.58 | 27.06 | 0.75 | 0.39 |
|  | A1 | 92645 | 113.61 | 51.35 | 33.68 | 7.29 | 17.51 | 5.11 | 3.09 | 3.31 | 2.73 |  | 66.21 | 41.99 | 9.97 | 6.62 | 3.04 | 0.29 |
|  | A1 | 94263 | 79.3 | 39.59 | 15.27 | 0.76 | 3.38 | 1.41 | 1.14 | 1.3 | 1 |  | 44.72 | 32.32 | 1.85 | 1.36 | 1.15 | 0.15 |
|  | A1 | 107454 | 78.46 | 24.61 | 29.2 | 4.49 | 17.57 | 2.73 | 2.29 | 1.54 | 2.13 |  | 44.09 | 29.85 | 8.26 | 8.11 | 1.99 | 0.4 |
|  | A1 | 110047 | 275.93 | 181.16 | 71.7 | 27.47 | 21.52 | 24.26 | 18.77 | 19.91 | 18.76 |  | 176.26 | 102.2 | 24.41 | 2.98 | 19.15 | 0.66 |
|  | A1 | 110888 | 114.08 | 82.36 | 89.29 | 17.88 | 22.31 | 10.14 | 40.66 | 36.23 | 42.48 |  | 95.24 | 16.68 | 16.77 | 6.16 | 39.79 | 3.21 |
| GA | G1 | 90046 | 350.32 | 139.91 | 143.98 | 3.91 | 19.78 | 1.12 | 0 | 0.17 | 0.1 |  | 211.4 | 120.32 | 8.27 | 10.06 | 0.09 | 0.09 |
|  | G1 | 91855 | 34.37 | 31.9 | 8.37 | 76.44 | 63.78 | 73.16 | 31.01 | 34.07 | 22.88 |  | 24.88 | 14.35 | 71.13 | 6.57 | 29.32 | 5.78 |
|  | G1 | 105030 | 455.01 | 230.11 | 131.22 | 22.97 | 85.17 | 17.99 | 11.28 | 13.95 | 12.25 |  | 272.11 | 165.93 | 42.04 | 37.43 | 12.49 | 1.36 |
| SB | S8/S53 | 34453 | 92.03 | 43.99 | 81.14 | 15.78 | 10.97 | 1.67 | 3.07 | 2.03 | 2.37 |  | 72.39 | 25.19 | 9.47 | 7.17 | 2.49 | 0.53 |
|  | S8/S53 | 37389 | 5.96 | 4.3 | 3.38 | 0.84 | 1.04 | 0.75 | 0.28 | 0.38 | 0.45 |  | 4.55 | 1.3 | 0.87 | 0.15 | 0.37 | 0.09 |
|  | S8/S53 | 70312 | 437.02 | 609.38 | 662.56 | 367.48 | 357.49 | 714.78 | 1770.97 | 1557.64 | 1727.13 |  | 569.65 | 117.9 | 479.92 | 203.46 | 1685.25 | 112.66 |
|  | S8/S53 | 72506 | 22.96 | 35.63 | 88.03 | 4.03 | 4.61 | 2.19 | 2.32 | 5.05 | 3.45 |  | 48.87 | 34.5 | 3.61 | 1.27 | 3.6 | 1.37 |
|  | S8/S53 | 72659 | 0 | 9.43 | 1.77 | 43.35 | 123.16 | 31.71 | 3.07 | 5.53 | 5.86 |  | 3.73 | 5.01 | 66.07 | 49.78 | 4.82 | 1.52 |
|  | S8/S53 | 75846 | 31.74 | 24.84 | 12.8 | 96.73 | 53.85 | 238.35 | 493.81 | 355.45 | 430.95 |  | 23.13 | 9.59 | 129.64 | 96.56 | 426.74 | 69.28 |
|  | S8/S53 | 83794 | 7.77 | 3.85 | 1.26 | 4.74 | 4.65 | 3.84 | 4.34 | 2.6 | 3.13 |  | 4.29 | 3.28 | 4.41 | 0.5 | 3.36 | 0.89 |
|  | S8/S53 | 84465 | 47.65 | 34.04 | 13.3 | 5.03 | 11.73 | 3.24 | 0.51 | 0.55 | 0.48 |  | 31.66 | 17.3 | 6.67 | 4.48 | 0.51 | 0.03 |
|  | S8/S53 | 91795 | 33.89 | 43.06 | 15.4 | 43.3 | 18.93 | 55.17 | 64.92 | 53.12 | 68.42 |  | 30.79 | 14.09 | 39.13 | 18.47 | 62.15 | 8.02 |
|  | S8/S53 | 109122 | 1.73 | 3 | 1.97 | 3.99 | 3.27 | 1.82 | 1.47 | 1.26 | 1.51 |  | 2.23 | 0.68 | 3.03 | 1.1 | 1.41 | 0.14 |
| SC | S10 | 68068 | 404.65 | 183.18 | 103.67 | 34.44 | 71.73 | 33.82 | 41.19 | 33.39 | 33.93 |  | 230.5 | 155.97 | 46.67 | 21.71 | 36.17 | 4.36 |
|  | S10 | 74336 | 0 | 0.83 | 1.09 | 2.54 | 6.64 | 5.03 | 25.75 | 29.52 | 30.45 |  | 0.64 | 0.57 | 4.74 | 2.07 | 28.57 | 2.49 |
|  | S10 | 77689 | 168.22 | 91.6 | 38.23 | 35.92 | 126.88 | 26.59 | 4.02 | 5.64 | 4.63 |  | 99.35 | 65.34 | 63.13 | 55.4 | 4.76 | 0.82 |
|  | S10 | 75070 | 847.96 | 399.84 | 231.33 | 24.49 | 167.51 | 20.51 | 19.98 | 25.76 | 18.51 |  | 493.04 | 318.71 | 70.83 | 83.74 | 21.41 | 3.83 |
|  | S10 | 90471 | 3.03 | 3.5 | 9.19 | 3.69 | 2.33 | 2.95 | 4.45 | 4.99 | 4.27 |  | 5.24 | 3.43 | 2.99 | 0.68 | 4.57 | 0.37 |
|  | S10 | 99840 | 23.28 | 28.57 | 24.24 | 41.15 | 42.26 | 46.37 | 58.86 | 64.42 | 53.36 |  | 25.36 | 2.82 | 43.26 | 2.75 | 58.88 | 5.53 |
|  | S10 | 103135 | 131.96 | 156.66 | 161.04 | 87.58 | 76.28 | 178.67 | 606.18 | 431.97 | 505.04 |  | 149.89 | 15.68 | 114.18 | 56.14 | 514.4 | 87.48 |
|  | S10 | 106874 | 4.51 | 0.98 | 2.56 | 4.94 | 3.78 | 7.39 | 48.78 | 32.74 | 46.76 |  | 2.68 | 1.77 | 5.37 | 1.84 | 42.76 | 8.74 |
|  | S10 | 109759 | 23.94 | 16.79 | 7.77 | 107.76 | 105.81 | 130.23 | 465.05 | 286.25 | 329.61 |  | 16.17 | 8.1 | 114.6 | 13.57 | 360.3 | 93.27 |
|  | S28 | 66250 | 116.72 | 79.93 | 29.05 | 56.72 | 86.87 | 44.66 | 21.8 | 23.75 | 17.27 |  | 75.23 | 44.02 | 62.75 | 21.74 | 20.94 | 3.33 |
|  | S28 | 70079 | 26.23 | 9.47 | 4.97 | 10.64 | 14.35 | 8.17 | 3.06 | 3.9 | 2.93 |  | 13.55 | 11.2 | 11.05 | 3.11 | 3.29 | 0.53 |
|  | S28 | 76675 | 8.24 | 15.29 | 0 | 4.63 | 4.68 | 3.43 | 1.61 | 2.52 | 1.9 |  | 7.84 | 7.65 | 4.25 | 0.71 | 2.01 | 0.46 |
|  | S28 | 108506 | 0 | 0 | 1.3 | 4.64 | 4.32 | 9.89 | 14.02 | 12.82 | 14.58 |  | 0.43 | 0.75 | 6.28 | 3.13 | 13.81 | 0.9 |
| PC | S51 | 49854 | 0 | 0 | 0 | 3.71 | 8.77 | 3.91 | 8.18 | 6.16 | 4.47 |  | 0 | 0 | 5.47 | 2.87 | 6.27 | 1.86 |
| MC | M14 | 59604 | 0 | 0 | 2.59 | 8.31 | 2.38 | 18.34 | 33.06 | 38.66 | 27.06 |  | 0.86 | 1.49 | 9.68 | 8.07 | 32.93 | 5.8 |
| MA | M3 | 38371 | 74.08 | 26.8 | 9.25 | 24.02 | 77.44 | 17.65 | 8.84 | 6.89 | 6.81 |  | 36.71 | 33.53 | 39.7 | 32.83 | 7.51 | 1.15 |
| MA | M35 | 39241 | 10.22 | 0 | 0 | 0 | 2.5 | 0.41 | 0.14 | 0.24 | 0.07 |  | 3.41 | 5.9 | 0.97 | 1.34 | 0.15 | 0.08 |
| MH | M28 | 95621 | 9.34 | 1.8 | 2.36 | 0.76 | 2.61 | 1.37 | 1.13 | 2.04 | 1.81 |  | 4.5 | 4.2 | 1.58 | 0.94 | 1.66 | 0.47 |
| MH | M28 | 65261 | 58.25 | 42.55 | 18.17 | 10.91 | 17.23 | 6.8 | 20.79 | 15.89 | 17.22 |  | 39.66 | 20.2 | 11.65 | 5.25 | 17.97 | 2.53 |
| MA | M43 | 76021 | 13.99 | 19.05 | 22.71 | 34.93 | 15.49 | 39.73 | 21.78 | 22.91 | 21.03 |  | 18.58 | 4.38 | 30.05 | 12.83 | 21.91 | 0.95 |

**Table S3** Transcription data for 39 secreted peptidases of *Zymoseptoria tritici* listed according to the MEROPS classification system into clans and families. Prot. ID corresponds to protein identification number from the Joint Genome Institute (JGI). For each of the three lifecycle stages (biotroph, necrotroph and saprotroph) RNA-seq reads of biological replicates are represented as mean and standard deviation (SD) of Z-score transformed TMM Normalised log CPM values. For the two transitions (biotroph to necrotroph and necrotroph to saprotroph) log fold change (log FC), fold change (FC) and Benjamin-Hochberg false discovery rates adjusted p-values (FDR) are shown. Fold change ≥5x between life cycle stages are indicated in purple. Significant FDR < 0.05 are indicated by *.

| MEROPS | MEROPS Family | JGI | 7 days post inoculation | | 14 days post inoculation | | 28 days post inoculation | | 7dpi_14dpi | | | 14dpi_28dpi | | |
| --- | --- | --- | --- | --- | --- | --- | --- | --- | --- | --- | --- | --- | --- | --- |
| CLAN |  | Prot ID | Mean Z-score | SD | Mean Z-score | SD | Mean Z-score | SD | logFC | FC | FDR | logFC | FC | FDR |
| AA | A1 | 92644 | 1.05 | 0.33 | 0.08 | 0.48 | -1.14 | 0.21 | -2.98 | 7.90 | 0.00* | -5.0 | 31.30 | 0.00* |
|  | A1 | 92645 | 1.19 | 0.46 | -0.23 | 0.47 | -0.95 | 0.07 | -2.75 | 6.70 | 0.00* | -1.7 | 3.29 | 0.00* |
|  | A1 | 94263 | 1.26 | 0.48 | -0.56 | 0.43 | -0.70 | 0.07 | -4.75 | 26.82 | 0.00* | -0.5 | 1.41 | 0.37 |
|  | A1 | 107454 | 1.14 | 0.45 | -0.20 | 0.68 | -0.94 | 0.15 | -2.46 | 5.50 | 0.00* | -2.0 | 4.04 | 0.00* |
|  | A1 | 110047 | 1.25 | 0.67 | -0.54 | 0.10 | -0.71 | 0.03 | -2.87 | 7.31 | 0.00* | -0.4 | 1.30 | 0.28 |
|  | A1 | 110888 | 1.08 | 0.22 | -1.14 | 0.49 | 0.06 | 0.11 | -2.53 | 5.79 | 0.00* | 1.2 | 2.38 | 0.00* |
| GA | G1 | 90046 | 1.13 | 0.17 | -0.03 | 0.44 | -1.10 | 0.21 | -4.76 | 27.01 | 0.00* | -6.2 | 73.74 | 0.00* |
|  | G1 | 91855 | -0.76 | 1.20 | 1.00 | 0.12 | -0.24 | 0.31 | 1.47 | 2.77 | 0.01* | -1.3 | 2.46 | 0.00* |
|  | G1 | 105030 | 1.18 | 0.46 | -0.27 | 0.60 | -0.91 | 0.07 | -2.71 | 6.53 | 0.00* | -1.8 | 3.37 | 0.00* |
| SB | S8/S53 | 34453 | 1.18 | 0.25 | -0.30 | 0.74 | -0.88 | 0.14 | -2.95 | 7.74 | 0.00* | -1.9 | 3.83 | 0.01* |
|  | S8/S53 | 37389 | 1.24 | 0.28 | -0.27 | 0.15 | -0.98 | 0.20 | -2.44 | 5.44 | 0.01* | -1.2 | 2.25 | 0.04* |
|  | S8/S53 | 70312 | -0.44 | 0.30 | -0.80 | 0.60 | 1.24 | 0.12 | -0.27 | 1.21 | 0.67 | 1.8 | 3.47 | 0.00* |
|  | S8/S53 | 72506 | 1.26 | 0.51 | -0.65 | 0.29 | -0.61 | 0.28 | -3.80 | 13.89 | 0.00* | 0.0 | 1.02 | 1.00 |
|  | S8/S53 | 72659 | -0.84 | 1.21 | 0.94 | 0.31 | -0.10 | 0.15 | 4.06 | 16.68 | 0.00* | -3.8 | 13.83 | 0.00* |
|  | S8/S53 | 75846 | -1.11 | 0.35 | 0.03 | 0.55 | 1.07 | 0.13 | 2.45 | 5.45 | 0.00* | 1.7 | 3.25 | 0.00* |
|  | S8/S53 | 83794 | -0.16 | 1.86 | 0.30 | 0.21 | -0.15 | 0.54 | -0.01 | 1.00 | 1.00 | -0.4 | 1.32 | 0.39 |
|  | S8/S53 | 84465 | 1.04 | 0.38 | 0.13 | 0.37 | -1.17 | 0.03 | -2.29 | 4.88 | 0.00* | -3.7 | 12.66 | 0.00* |
|  | S8/S53 | 91795 | -0.62 | 1.02 | -0.26 | 1.03 | 0.88 | 0.26 | 0.31 | 1.24 | 0.70 | 0.6 | 1.54 | 0.13 |
|  | S8/S53 | 109122 | 0.14 | 0.76 | 0.79 | 1.04 | -0.93 | 0.28 | 0.37 | 1.29 | 0.85 | -1.1 | 2.11 | 0.04* |
| SC | S10 | 68068 | 1.18 | 0.77 | -0.52 | 0.48 | -0.66 | 0.14 | -2.32 | 4.98 | 0.00* | -0.4 | 1.30 | 0.36 |
|  | S10 | 74336 | -1.07 | 0.92 | 0.11 | 0.22 | 0.96 | 0.03 | 2.84 | 7.18 | 0.02* | 2.6 | 6.17 | 0.00* |
|  | S10 | 77689 | 0.80 | 0.54 | 0.40 | 0.59 | -1.20 | 0.11 | -0.66 | 1.58 | 0.45 | -3.7 | 13.40 | 0.00* |
|  | S10 | 75070 | 1.16 | 0.44 | -0.36 | 0.77 | -0.80 | 0.11 | -2.79 | 6.93 | 0.00* | -1.8 | 3.37 | 0.01* |
|  | S10 | 90471 | 0.39 | 1.47 | -0.82 | 0.53 | 0.43 | 0.19 | -0.78 | 1.71 | 0.39 | 0.6 | 1.47 | 0.19 |
|  | S10 | 99840 | -1.18 | 0.28 | 0.11 | 0.20 | 1.07 | 0.24 | 0.75 | 1.68 | 0.12 | 0.4 | 1.34 | 0.22 |
|  | S10 | 103135 | -0.37 | 0.12 | -0.86 | 0.59 | 1.23 | 0.23 | -0.42 | 1.34 | 0.49 | 2.2 | 4.45 | 0.00* |
|  | S10 | 106874 | 0.95 | 0.94 | -0.90 | 0.70 | -0.05 | 0.23 | -1.80 | 3.49 | 0.00* | 0.6 | 1.56 | 0.15 |
|  | S10 | 109759 | -1.21 | 0.41 | 0.19 | 0.09 | 1.02 | 0.18 | 2.79 | 6.92 | 0.00* | 1.6 | 3.10 | 0.00* |
|  | S28 | 66250 | 0.60 | 1.09 | 0.45 | 0.51 | -1.05 | 0.25 | -0.28 | 1.21 | 0.73 | -1.6 | 3.03 | 0.00* |
|  | S28 | 70079 | 0.54 | 1.17 | 0.50 | 0.39 | -1.04 | 0.20 | -0.32 | 1.25 | 0.72 | -1.7 | 3.35 | 0.00* |
|  | S28 | 76675 | -0.23 | 1.94 | 0.31 | 0.09 | -0.08 | 0.12 | -0.94 | 1.91 | 0.44 | -1.1 | 2.10 | 0.01* |
|  | S28 | 108506 | -1.18 | 0.87 | 0.41 | 0.19 | 0.77 | 0.03 | 3.98 | 15.76 | 0.00* | 1.1 | 2.10 | 0.01* |
| PC | S51 | 49854 | -1.32 | 0.00 | 0.61 | 0.21 | 0.71 | 0.13 | 7.58 | 191.88 | 0.01* | 0.3 | 1.23 | 0.71 |
| MC | M14 | 59604 | -1.12 | 0.85 | 0.25 | 0.40 | 0.87 | 0.07 | 3.61 | 12.20 | 0.03* | 1.7 | 3.18 | 0.00* |
| MA | M3 | 38371 | 0.38 | 1.13 | 0.55 | 0.84 | -0.94 | 0.17 | 0.11 | 1.08 | 0.93 | -2.4 | 5.35 | 0.00* |
| MA | M35 | 39241 | 0.02 | 1.65 | 0.17 | 1.06 | -0.19 | 0.23 | NA | NA | NA | NA | NA | NA |
| MH | M28 | 95621 | 0.78 | 1.27 | -0.53 | 0.89 | -0.25 | 0.42 | -1.59 | 3.01 | 0.17 | 0.2 | 1.15 | 0.88 |
| MH | M28 | 65261 | -0.90 | 0.55 | -0.33 | 0.24 | 1.22 | 0.16 | 1.02 | 2.03 | 0.35 | 2.9 | 7.58 | 0.00* |
| MA | M43 | 76021 | -0.61 | 0.70 | 0.58 | 1.55 | 0.03 | 0.13 | 0.69 | 1.61 | 0.34 | -0.5 | 1.4 | 0.26 |

**Table S4** Table displaying the average dN/dS (Ka/Ks) ratios for the entire gene, for 39 secreted and 39 non-secreted peptidases calculated by pairwise sequence comparisons among 29 *Z. tritici* isolates using DnaSP.

| **Secreted Peptidases** | | **Non- Secreted Peptidases** | |
| --- | --- | --- | --- |
| JGI | Ka / Ks | JGI | Ka / Ks |
| Prot ID |  | Prot ID |  |
| 92644 | 2.82 | 34175 | 0.07 |
| 92645 | 0.31 | 34306 | 0.82 |
| 94263 | 0.10 | 35862 | 0.42 |
| 107454 | 19.35 | 37519 | 0.49 |
| 110047 | 0.24 | 41275 | 0.01 |
| 110888 | 0.14 | 42932 | 0.22 |
| 90046 | 0.16 | 45120 | 0.00 |
| 91855 | 0.17 | 45160 | 0.05 |
| 105030 | 0.68 | 47075 | 0.28 |
| 34453 | 0.35 | 47386 | 0.88 |
| 37389 | 0.22 | 49589 | 0.08 |
| 70312 | 0.04 | 49727 | 0.46 |
| 72506 | 0.05 | 59096 | 0.27 |
| 72659 | 0.04 | 59247 | 0.12 |
| 75846 | 0.03 | 60951 | 0.01 |
| 83794 | 0.14 | 65568 | 0.03 |
| 84465 | 0.54 | 68734 | 0.99 |
| 91795 | 0.21 | 70030 | 0.08 |
| 109122 | 0.09 | 70588 | 0.01 |
| 68068 | 1.29 | 71911 | 0.39 |
| 74336 | 0.06 | 75432 | 0.01 |
| 75070 | 0.07 | 75573 | 0.23 |
| 77689 | 0.17 | 77395 | 0.02 |
| 90471 | 0.15 | 83488 | 0.05 |
| 99840 | 0.18 | 87626 | 0.12 |
| 103135 | 0.05 | 88401 | 0.04 |
| 106874 | 0.09 | 91725 | 0.17 |
| 109759 | 0.05 | 92612 | 0.08 |
| 66250 | 0.18 | 93284 | 0.31 |
| 70079 | 0.44 | 93286 | 0.66 |
| 76675 | 0.25 | 99102 | 0.00 |
| 108506 | 0.06 | 101415 | 0.04 |
| 49854 | 0.18 | 101437 | 0.02 |
| 59604 | 0.04 | 104721 | 0.02 |
| 38371 | 4.64 | 106357 | 0.24 |
| 39241 | 1.74 | 108793 | 0.25 |
| 95621 | 0.11 | 108985 | 0.08 |
| 65261 | 0.03 | 110366 | 0.08 |
| 76021 | 0.01 | 1078351 | 0.16 |
|  | |  | |
| Average | 0.91 | Average | 0.21 |
| Median | 0.16 | Median | 0.08 |

**Table S5** Likelihood ratio test to identify signatures of selection in genes encoding secreted peptidases in *Zymoseptoria sp*. Selection model (M8) was compared against the neutral model (M7). ProtID; the protein identification number from the Joint Genome Institute (JGI). Significant p values ≤ 0.01 are indicated by *.

| MEROPS | MEROPS Family | JGI | LnL | LnL |  |  |  |  |
| --- | --- | --- | --- | --- | --- | --- | --- | --- |
| CLAN |  | Prot ID | M7 | M8 | Δlnl | 2*Δlnl | P value | Selection |
| AA | A1 | 92644 | -3540.04 | -3473.81 | 66.24 | 132.47 | 0.00* | Diversifying |
|  | A1 | 92645 | -2903.39 | -2883.59 | 19.8 | 39.59 | 0.00* | Diversifying |
|  | A1 | 94263 | -4620.61 | -4548.96 | 71.65 | 143.3 | 0.00* | Diversifying |
|  | A1 | 107454 | -2917.16 | -2909.39 | 7.77 | 15.54 | 0.00* | Diversifying |
|  | A1 | 110047 | -3050.5 | -3043.47 | 7.03 | 14.05 | 0.00* | Diversifying |
|  | A1 | 110888 | -57621.87 | -57572.37 | 49.5 | 98.99 | 0.00* | Diversifying |
| GA | G1 | 90046 | -1869.94 | -1867.77 | 2.17 | 4.34 | 0.11 | Neutral |
|  | G1 | 91855 | -1918.92 | -1902.47 | 16.45 | 32.9 | 0.00* | Diversifying |
|  | G1 | 105030 | -5465.4 | -5449.89 | 15.51 | 31.02 | 0.00* | Diversifying |
| SB | S8/S53 | 34453 | -2840.07 | -2831.02 | 9.05 | 18.09 | 0.00* | Diversifying |
|  | S8/S53 | 37389 | -4658.81 | -4642.14 | 16.66 | 33.32 | 0.00* | Diversifying |
|  | S8/S53 | 70312 | -2834.26 | -2817.49 | 16.77 | 33.54 | 0.00* | Diversifying |
|  | S8/S53 | 72506 | -20976.32 | -20973.03 | 3.29 | 6.58 | 0.04 | Neutral |
|  | S8/S53 | 72659 | -2716.39 | -2703.92 | 12.47 | 24.93 | 0.00* | Diversifying |
|  | S8/S53 | 75846 | -63831.86 | -63822.23 | 9.63 | 19.26 | 0.00* | Diversifying |
|  | S8/S53 | 83794 | -11897.76 | -11885.36 | 12.39 | 24.79 | 0.00* | Diversifying |
|  | S8/S53 | 84465 | -5243.8 | -5241.8 | 2.01 | 4.02 | 0.13 | Neutral |
|  | S8/S53 | 91795 | -2858.66 | -2856.54 | 2.13 | 4.26 | 0.12 | Neutral |
|  | S8/S53 | 109122 | -5762.31 | -5758.79 | 3.52 | 7.03 | 0.03 | Neutral |
| SC | S10 | 68068 | -4035.83 | -3999.48 | 36.35 | 72.69 | 0.00* | Diversifying |
|  | S10 | 74336 | -83547.62 | -83456.25 | 91.36 | 182.73 | 0.00* | Diversifying |
|  | S10 | 75070 | -4561.05 | -4545.08 | 15.97 | 31.95 | 0.00* | Diversifying |
|  | S10 | 77689 | -4179.02 | -4164.93 | 14.08 | 28.17 | 0.00* | Diversifying |
|  | S10 | 90471 | -35574.75 | -35560.82 | 13.93 | 27.86 | 0.00* | Diversifying |
|  | S10 | 99840 | -5137.93 | -5004.71 | 133.23 | 266.45 | 0.00* | Diversifying |
|  | S10 | 103135 | -9117.78 | -9108.88 | 8.89 | 17.79 | 0.00* | Diversifying |
|  | S10 | 106874 | -3391.63 | -3386.44 | 5.2 | 10.39 | 0.01* | Diversifying |
|  | S10 | 109759 | -3092.68 | -3092.68 | 0 | 0 | 1 | Neutral |
|  | S28 | 66250 | -3560.89 | -3556.99 | 3.9 | 7.8 | 0.02 | Neutral |
|  | S28 | 70079 | -49810.74 | -49808.75 | 1.99 | 3.98 | 0.14 | Neutral |
|  | S28 | 76675 | -17530.16 | -17522.16 | 7.99 | 15.99 | 0 | Diversifying |
|  | S28 | 108506 | -2950.48 | -2949.83 | 0.65 | 1.3 | 0.52 | Neutral |
| PC | S51 | 49854 | -1816.41 | -1815.24 | 1.17 | 2.33 | 0.31 | Neutral |
| MC | M14 | 59604 | -47669.33 | -47647.24 | 22.09 | 44.18 | 0 | Diversifying |
| MA | M3 | 38371 | -4031.31 | -4018.76 | 12.55 | 25.1 | 0.00* | Diversifying |
| MA | M35 | 39241 | -2094.4 | -2082.27 | 12.13 | 24.25 | 0.00* | Diversifying |
| MH | M28 | 95621 | -31887.72 | -31826.79 | 60.93 | 121.86 | 0 | Diversifying |
| MH | M28 | 65261 | -37141 | -37086.54 | 54.46 | 108.92 | 0 | Diversifying |
| MA | M43 | 76021 | -2490.01 | -2487.52 | 2.5 | 5 | 0.08 | Neutral |

**Table S6** PAML based likelihood ratio tests to identify differential selection in the branch leading to *Z. tritici* compared to the other branches in the phylogeny. The branch model was compared against the M0 model that estimates a single omega value for all branches. Four out of 39 genes encoding secreted peptidases showed significantly elevated rates of evolution for the *Z. tritici* lineage (significant p values ≤ 0.01 are indicated by *). Two of them encode aspartic peptidases in the A1 family.

| MEROPS | MEROPS Family | JGI ProtID | LnL | LnL | 2ΔLnL | p value | Omega(fg) | Omega(bg) | Selection |
| --- | --- | --- | --- | --- | --- | --- | --- | --- | --- |
| Clan |  |  | BM(np=3) | M0(np=2) |  | df=1 |  |  |  |
| AA | A1 | 92644 | -3590.69 | -3590.70 | 0.02 | 0.89 | 0.67 | 0.70 |  |
|  | A1 | 92645 | -2919.03 | -2922.54 | 7.02 | 0.01* | 0.89 | 0.35 | Relaxed purifying |
|  | A1 | 94263 | -3811.54 | -3811.66 | 0.24 | 0.62 | 0.16 | 0.18 |  |
|  | A1 | 107454 | -2951.21 | -2952.12 | 1.82 | 0.18 | 0.26 | 0.44 |  |
|  | A1 | 110047 | -3065.66 | -3073.15 | 14.98 | 0.00* | 0.83 | 0.26 | Relaxed purifying |
|  | A1 | 110888 | -4693.69 | -4693.89 | 0.42 | 0.52 | 0.23 | 0.19 |  |
| GA | G1 | 90046 | -1932.13 | -1932.17 | 0.08 | 0.78 | 0.36 | 0.30 |  |
|  | G1 | 91855 | -1916.13 | -1916.25 | 0.23 | 0.63 | 0.24 | 0.33 |  |
|  | G1 | 105030 | -2367.08 | -2369.92 | 5.68 | 0.01* | 4.68 | 0.71 | Relaxed purifying |
| SB | S8/S53 | 34453 | -2849.63 | -2849.73 | 0.20 | 0.65 | 0.07 | 0.09 |  |
|  | S8/S53 | 37389 | -4806.50 | -4806.62 | 0.23 | 0.63 | 0.26 | 0.23 |  |
|  | S8/S53 | 70312 | -2849.45 | -2850.09 | 1.28 | 0.26 | 0.13 | 0.04 |  |
|  | S8/S53 | 72506 | -4573.20 | -4573.22 | 0.03 | 0.86 | 0.07 | 0.07 |  |
|  | S8/S53 | 72659 | -2618.22 | -2618.69 | 0.92 | 0.34 | 0.03 | 0.06 |  |
|  | S8/S53 | 75846 | -5679.26 | -5679.27 | 0.03 | 0.87 | 0.03 | 0.03 |  |
|  | S8/S53 | 83794 | -4235.32 | -4235.33 | 0.02 | 0.89 | 0.22 | 0.20 |  |
|  | S8/S53 | 84465 | -5346.75 | -5349.21 | 4.92 | 0.27 | 0.15 | 0.25 |  |
|  | S8/S53 | 91795 | -2906.04 | -2906.06 | 0.05 | 0.83 | 0.14 | 0.20 |  |
|  | S8/S53 | 109122 | -2768.16 | -2768.24 | 0.16 | 0.69 | 0.14 | 0.17 |  |
| SC | S10 | 68068 | -4120.69 | -4120.70 | 0.01 | 0.96 | 0.40 | 0.39 |  |
|  | S10 | 74336 | -8645.92 | -8645.97 | 0.10 | 0.75 | 0.08 | 0.10 |  |
|  | S10 | 77689 | -4306.34 | -4307.03 | 1.37 | 0.24 | 0.31 | 0.23 |  |
|  | S10 | 75070 | -4508.50 | -4509.92 | 2.83 | 0.09 | 0.15 | 0.08 |  |
|  | S10 | 90471 | -5341.27 | -5349.87 | 17.21 | 0.00* | 0.38 | 0.17 | Diversifying |
|  | S10 | 99840 | -4411.91 | -4413.48 | 3.14 | 0.08 | 0.08 | 0.17 |  |
|  | S10 | 103135 | -3452.45 | -3452.47 | 0.04 | 0.84 | 0.10 | 0.05 |  |
|  | S10 | 106874 | -3405.04 | -3405.42 | 0.76 | 0.38 | 0.18 | 0.28 |  |
|  | S10 | 109759 | -3106.24 | -3108.42 | 4.35 | 0.04 | 0.04 | 0.05 |  |
|  | S28 | 66250 | -3594.99 | -3595.46 | 0.95 | 0.33 | 0.44 | 0.27 |  |
|  | S28 | 70079 | -3810.20 | -3811.17 | 1.93 | 0.17 | 0.41 | 0.27 |  |
|  | S28 | 76675 | -3684.12 | -3684.67 | 1.09 | 0.30 | 0.28 | 0.38 |  |
|  | S28 | 108506 | -2956.97 | -2958.06 | 2.18 | 0.14 | 0.03 | 0.12 |  |
| PC | S51 | 49854 | -1823.61 | -1825.22 | 3.23 | 0.07 | 0.17 | 0.39 |  |
| MC | M14 | 59604 | -3623.37 | -3623.85 | 0.96 | 0.33 | 0.00 | 0.07 |  |
| MA | M3 | 38371 | -4051.36 | -4051.36 | 0.00 | 0.98 | 0.31 | 0.31 |  |
| MA | M35 | 39241 | -2104.78 | -2105.79 | 2.02 | 0.16 | 0.42 | 0.88 |  |
| MH | M28 | 95621 | -5697.22 | -5697.79 | 1.14 | 0.29 | 0.10 | 0.16 |  |
| MH | M28 | 65261 | -5076.30 | -5076.73 | 0.86 | 0.35 | 0.08 | 0.04 |  |
| MA | M43 | 76021 | -2502.09 | -2502.21 | 0.24 | 0.65 | 0.21 | 0.17 |  |

**Table S7** Likelihood ratio test to identify signatures of selection on the sister genes encoding aspartic endo-peptidases, after each duplication event. “Branch” corresponds to the branch number indicated in Fig. 3. Colors correspond to the two MEROPS families A1 (orange) and G1 (green). Significant p values ≤ 0.01 are indicated by *.

|  |  |  |  |  |  |  |  |  |  |  |  |  |  |  |  |  |
| --- | --- | --- | --- | --- | --- | --- | --- | --- | --- | --- | --- | --- | --- | --- | --- | --- |
|  |  | **LnL** | **LnL** | **ΔLnL** | **2ΔLnL** | **p value** |  | **ω** | **ω** |  | **LnL** | **2ΔLnL** | **p value** |  | **Evolution** | Model |
| **Branch** | **Gene** | **BM(np=3)** | **M0(np=2)** |  |  | **df=1** |  | **foreground** | **background** |  | BM: **ω** =1 | | **df=1** |  |  |  |
|  |  |  |  |  |  |  |  |  |  |  |  |  |  |  |  |  |
| 1 |  | **-67459.197** | **-67475.415** | **16.218** | **32.436** | **< 0.001*** |  | Infinite | 0.4969 |  | -67467.729 | 17.065 | < 0.001* |  | Diversifying |  |
| 2 |  | **-67465.162** | **-67475.415** | **10.253** | **20.506** | **< 0.001*** |  | 5.0151 | 0.4929 |  | -67468.635 | 6.947 | 0.008* |  | Diversifying | EAC |
| 3 | 90046 | **-67454.997** | **-67475.415** | **20.418** | **40.837** | **< 0.001*** |  | 3.0492 | 0.4912 |  | -67460.950 | 11.907 | 0.001* |  | Diversifying | EAC |
| 4 | 105030 | **-67457.429** | **-67475.415** | **17.985** | **35.971** | **< 0.001*** |  | 2.2034 | 0.4907 |  | -67461.386 | 7.913 | 0.005* |  | Diversifying | EAC |
| 5 | 91855 | **-67457.917** | **-67475.415** | **17.498** | **34.996** | **< 0.001*** |  | 1.9825 | 0.4904 |  | -67461.242 | 6.649 | 0.010* |  | Diversifying | EAC |
|  |  |  |  |  |  |  |  |  |  |  |  |  |  |  |  |  |
| 6 |  | **-67464.363** | **-67475.415** | **11.052** | **22.103** | **< 0.001*** |  | Infinite | 0.4952 |  | -67471.084 | 13.441 | < 0.001* |  | Diversifying | EAC |
| 7 |  | **-67472.034** | **-67475.415** | **3.381** | **6.761** | **0.009*** |  | 55.3092 | 0.4966 |  | -67474.121 | 4.174 | 0.041 |  | Relaxed purifying | EAC |
| 8 |  | **-67464.956** | **-67475.415** | **10.459** | **20.917** | **< 0.001*** |  | 950.7079 | 0.4940 |  | -67470.561 | 11.209 | 0.001* |  | Diversifying | EAC |
| 9 | 92644 | **-67464.200** | **-67475.415** | **11.215** | **22.430** | **< 0.001*** |  | 1.1253 | 0.4919 |  | -67464.402 | 0.404 | 0.525 |  | Relaxed purifying | EAC |
| 10 | 110888 | **-67446.014** | **-67475.415** | **29.401** | **58.802** | **< 0.001*** |  | 2.6932 | 0.4877 |  | -67454.009 | 15.991 | < 0.001* |  | Diversifying | EAC |
| 11 |  | **-67470.474** | **-67475.415** | **4.941** | **9.882** | **0.002*** |  | 50.3607 | 0.4956 |  | -67473.251 | 5.554 | 0.018 |  | Relaxed purifying | EAC |
| 12 | 110047 | **-67458.974** | **-67475.415** | **16.441** | **32.882** | **< 0.001*** |  | 2.0125 | 0.4921 |  | -67462.345 | 6.742 | 0.009* |  | Diversifying | EAC |
| 13 | 92645 | **-67470.798** | **-67475.415** | **4.616** | **9.233** | **0.002*** |  | 0.8533 | 0.4941 |  | -67471.136 | 0.675 | 0.411 |  | Relaxed purifying | EAC |
| 14 |  | **-67458.574** | **-67476.415** | **17.841** | **35.683** | **< 0.001*** |  | 80.5007 | 0.4927 |  | -67466.685 | 16.222 | < 0.001* |  | Diversifying | EAC |
| 15 | 107454 | **-67472.861** | **-67475.415** | **2.554** | **5.109** | **0.024** |  | 0.7836 | 0.4950 |  | -67473.473 | 1.225 | 0.268 |  | Neutral | Conservation |
| 16 | 94263 | **-67464.557** | **-67475.415** | **10.858** | **21.716** | **< 0.001*** |  | 1.9132 | 0.4922 |  | -67466.470 | 3.826 | 0.050 |  | Relaxed purifying | NEO-F |
|  |  |  |  |  |  |  |  |  |  |  |  |  |  |  |  |  |
|  |  |  |  |  |  |  |  |  |  |  |  |  |  |  |  |  |

**Table S8:** Location of transposable elements (TE) relative to the secreted peptidases in the *Zymoseptoria tritici* genome. The presence of orthologous genes in the closely related species *Z. pseudotritici* and *Z. ardabiliae* is indicated with a “+”. TE-Family annotations is from Plissoneau et al. [52].

|  | **chromosome** | **nearest TE** | **TE-Family** | **orthologs in**  ***Z. pseudotritici*** | **orthologs in**  ***Z. ardabiliae*** |
| --- | --- | --- | --- | --- | --- |
| 92644 | 4 | 8051 bp downstream | DNA/Mutator | + | + |
| 92645 | 4 | 11274 bp downstream | DNA/Mutator | + | absent |
| 94263 | 7 | 5263 bp downstream | DNA/ Harbinger | + | + |
| 107454 | 1 | 30726bp downstream | LTR/Gypsy | + | + |
| 110047 | 7 | 6137bp upstream | DNA/ Harbinger | + | + |
| 110888 | 9 | 13566 bp downstream | LINE | + | absent |
| 90046 | 1 | 17244 bp upstream | Unknown | + | + |
| 91855 | 3 | 281 bp upstream | DNA/TcMar- TC1 | + | + |
| 105030 | 7 | 2500 bp downstream | DNA and LTRS | + | + |
| 34453 | 1 | 22403 bp downstream | RC/Helitron | + | + |
| 37389 | 2 | 15461 bp upstream | LTR /Gypsy | + | + |
| 70312 | 3 | 14950 bp downstream | LINE | + | + |
| 72506 | 5 | 3422 bp upstream | DNA/hAt | + | + |
| 72659 | 5 | 15832 bp downstream | RC/Helitron | + | + |
| 75846 | 9 | 5539bp downstream | DNA/TcMar- TC1 | + | + |
| 83794 | 1 | NA |  | + | + |
| 84465 | 2 | 2213 bp upstream |  | + | + |
| 91795 | 3 | 1477 kb downstream | DNA/hAt and unknown | + | + |
| 109122 | 4 | 11046 bp downstream | LTR/copia | + | + |
| 68068 | 2 | 62053 bp downstream | unknown | + | + |
| 74336 | 8 | 322 bp upstream | DNA | + | + |
| 75070 | 8 | NA |  | + | + |
| 77689 | 10 | NA |  | + | + |
| 90471 | 2 | 25621 bp upstream | DNA/TcMar- TC1 | + | + |
| 99840 | 4 | 19856kb upstream | mite | + | + |
| 103135 | 2 | NA |  | + | + |
| 106874 | 1 | 40 kb downstream | unknown | + | + |
| 109759 | 6 | 11p56bp upstream | LTR/copia | + | + |
| 66250 | 1 | 6996 bp upstream | DNA | + | + |
| 70079 | 3 | 15181 bp upstream | DNA/hAt | + | + |
| 76675 | 11 | 365 bpupstream | MITE | + | + |
| 108506 | 3 | 6881 bp downstream | DNA/Mutator | + | absent |
| 49854 | 11 | 413bp upstream | DNA/TcMar- TC1 | absent | + |
| 59604 | 6 | 24572 bp upstream | DNA/Mutator | + | + |
| 38371 | 3 | 24292 bpupstream | LTR/Gypsy | + | absent |
| 39241 | 3 | 804 bpupstream | RC/Helitron | absent | + |
| 95621 | 9 | 4877bp upstream | Mite | + | + |
| 65261 | 1 | 313954 bp downstream | RC/Helitron | + | + |
| 76021 | 10 | 25802 bp upstream | DNA/ Harbinger | + | + |

**Table S9:** Inferred recombination events using RDP4 for secreted peptidases. Protein IDs are according to reference isolate IPO323.

| Prot.ID. | no. of inferred recombinations |
| --- | --- |
| 92644 | 0 |
| 92645 | 0 |
| 107454 | 0 |
| 110047 | 0 |
| 90046 | 0 |
| 91855 | 0 |
| 105030 | 0 |
| 37389 | 0 |
| 70312 | 0 |
| 72659 | 0 |
| 109122 | 0 |
| 77689 | 0 |
| 106874 | 0 |
| 109759 | 0 |
| 49854 | 0 |
| 38371 | 0 |
| 39241 | 0 |
| 76021 | 0 |
| 72506 | 1 |
| 91795 | 1 |
| 68068 | 1 |
| 90471 | 1 |
| 66250 | 1 |
| 76675 | 1 |
| 94263 | 2 |
| 83794 | 2 |
| 84465 | 2 |
| 99840 | 2 |
| 103135 | 2 |
| 70079 | 2 |
| 108506 | 2 |
| 34453 | 3 |
| 75070 | 4 |
| 59604 | 5 |
| 65261 | 6 |
| 110888 | 7 |
| 75846 | 7 |
| 95621 | 8 |
| 74336 | 17 |


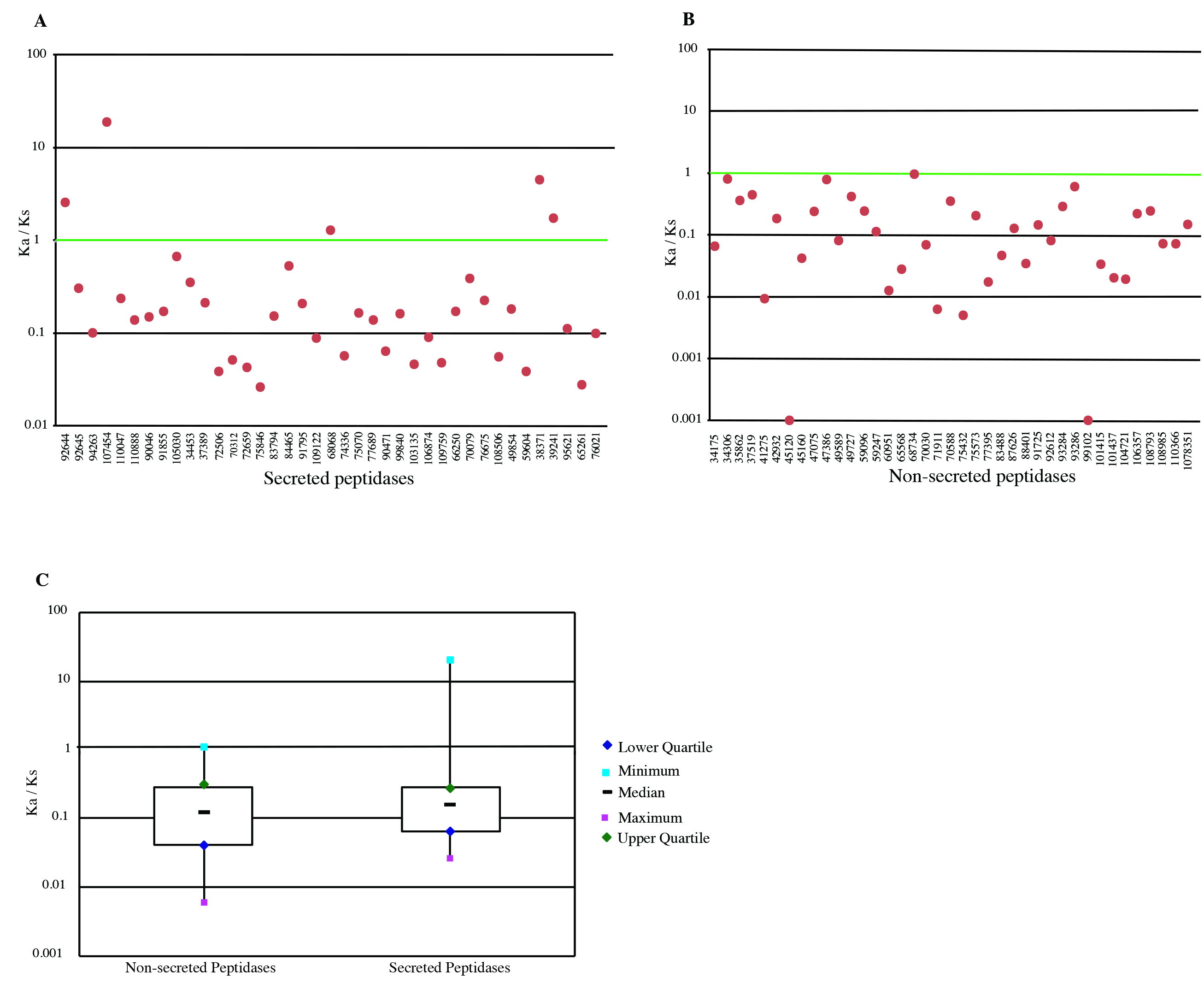


Fig. S1. Scatterplot representing the average dN/dS ratios of secreted (A) and non-secreted peptidases (B) calculated from pairwise sequence comparisons using DnaSP. The box & whiskers Plot (C) summarizing the average dN/dS ratios of secreted and non-secreted peptidases. The ratios obtained for the secreted peptidases are significantly higher then for the non-secreted peptidases (Wilcoxon rank-sum test; p = 0.033)

Fig. S2. Example of a phylogenetic tree used for the PAML “branch model” analyses to infer signatures of selection for the *Zymoseptoria tritici* lineage. Phylogenetic tree of gene ProtID-105030 encoding a secreted peptidase of MEROPS family G1. Included are 29 isolates of *Zymoseptoria tritici* (ST), five isolates of *Z. pseudotritici* (Zp) and four isolates of *Z. ardabiliae* (Za). The branch leading to *Z. tritici* (foreground branch) is indicated in red and has an ω value of 4.68, indicating an accelerated evolutionary rate compared to the background branches (ω = 0.70831).
